# A randomized, double-blind, placebo-controlled phase 2 study of intratympanic OTO-313 in patients with moderate to severe subjective tinnitus

**DOI:** 10.1007/s00405-023-08047-0

**Published:** 2023-06-21

**Authors:** Grant D. Searchfield, James M. Robinson, David Skarinsky, Yiwei Wang, Jeffery J. Anderson

**Affiliations:** 1 University of Auckland, Auckland, New Zealand; 2 Otonomy Inc., San Diego, CA USA

**Keywords:** Tinnitus, OTO-313, Gacyclidine, Randomized controlled trial, Placebo effect, NMDA antagonist

## Abstract

**Purpose:**

This was a randomized, double-blind, placebo-controlled Phase 2 study to evaluate the efficacy and safety of intratympanic OTO-313 in patients with subjective unilateral tinnitus.

**Methods:**

Patients with moderate to severe unilateral tinnitus of 2–12 months duration were enrolled. A single intratympanic injection of OTO-313 or placebo was administered to the affected ear and patients were evaluated during a 16-weeks follow-up period. Efficacy was assessed using the Tinnitus Functional Index (TFI), daily ratings of tinnitus loudness and annoyance, and Patient Global Impression of Change (PGIC).

**Results:**

Intratympanic administration of OTO-313 and placebo produced reductions in tinnitus with a similar percentage of TFI responders at Weeks 4, 8, 12, and 16. Reductions in daily ratings of tinnitus loudness and annoyance, and PGIC scores were also similar between OTO-313 and placebo groups. No significant differences in mean TFI scores between OTO-313 and placebo were observed for pre-specified strata regarding tinnitus duration (≥ 2 to ≤ 6 months and > 6 to ≤ 12 months) and TFI baseline scores (≥ 32 to ≤ 53 points and ≥ 54 to 100 points), although the results numerically favored OTO-313 in patients in the ≥ 2 to ≤ 6 months strata. These results also demonstrated an unexpectedly high placebo response particularly amongst patients with chronic tinnitus, despite training implemented to mitigate placebo response. OTO-313 was well-tolerated with a similar incidence of adverse events compared to placebo.

**Conclusions:**

OTO-313 did not demonstrate a significant treatment benefit relative to placebo due in part to a high placebo response. OTO-313 was safe and well-tolerated.

## Introduction

Tinnitus, often described as ringing in the ear, is a perception of a sound without corresponding external source. The condition is very common with prevalence rates of approximately 10% of the adult population in the United States reporting tinnitus in the past year [1]. Prevalence of tinnitus increases with age, peaking between 60 and 69 years of age, and is higher in individuals with a history of loud noise exposure from firearm usage, occupational noise, or recreational noise [2]. While some patients can cope with their tinnitus, many find it bothersome enough to seek medical treatment [1]. A significant proportion of patients experience moderate to severe tinnitus, which can negatively impact sleep, disrupt ability to focus, create feelings of distress, anxiety, and depression, and lower overall quality of life [3–7]. Current management focuses on education and counseling, sound therapy, use of hearing aids, and cognitive behavioral therapy [8]. These therapies focus primarily on modulation of patient’s attention and reactions to tinnitus rather than the sensation itself.

Tinnitus can occur in one or both ears and often arises as a consequence of acoustic trauma, barotrauma, or ototoxic drug exposure which may damage the cochlea. In animal models, excessive activation of the N-methyl-D-aspartic acid (NMDA) subtype of glutamate receptors at the level of the inner hair cell synapses with resulting deafferentation may be key in altering activity of the auditory nerve and generating subjective tinnitus [9, 10]. In animal models of acute tinnitus induced by traumatic noise or salicylate, intratympanic administration of an NMDA receptor antagonist was shown to reduce “tinnitus-like” behavior [10, 11]. These findings suggest that activation of cochlear NMDA receptors may be an important mechanism for generating tinnitus and that intratympanic NMDA receptor antagonists may have potential as a local cochlear treatment for tinnitus.

Gacyclidine is an NMDA-receptor antagonist [12] that has shown efficacy in animal models of tinnitus [9] and in a small open-label study in tinnitus patients [13]. OTO-313 is a lipid-based formulation of gacyclidine that provides sustained exposure to the cochlea after a single intratympanic injection [14]. Intratympanic delivery of drugs permits deposition over the round window membrane, allowing access to the inner ear for more localized delivery to the cochlea and less systemic drug exposure [15].

In a completed Phase 1/2 study in 35 patients with unilateral tinnitus [16], OTO-313 (0.32 mg) produced a greater number of responders on the Tinnitus Functional Index (TFI), defined by at least a 13-point reduction at 4 and 8 weeks after dosing, compared to placebo. Reductions in daily ratings of tinnitus loudness and annoyance favored OTO-313 compared to placebo, with strong correlations between change from baseline in TFI score and changes in tinnitus loudness, tinnitus annoyance, and the Patient Global Impression of Change (PGIC). The purpose of this study was to further evaluate the efficacy and safety of OTO-313 in a larger sample of tinnitus patients and to extend the follow-up period beyond 8 weeks to determine the optimal timeframe for response to OTO-313.

The initial clinical study evaluated patients with relatively acute tinnitus ranging from 1 to 6 months since onset based on the hypothesis that an NMDA receptor antagonist administered locally to the cochlea would be most effective in the acute period after onset and prior to tinnitus “centralization” [17, 18]. Because the initial study results, paradoxically, indicated better response in patients with onset from 3 to 6 months compared to 1–3 months, we expanded the timeframe of tinnitus duration to up to 12 months to further evaluate the treatment window. We stratified the randomization to ensure equal numbers of OTO-313 and placebo patients within the ≥ 2 months to ≤ 6 months and > 6 months to ≤ 12 months strata. Also in the initial study, eligible patients were required to have a minimum TFI score of 25 or higher. Study results demonstrated that patients who responded to OTO-313 had higher TFI scores, or more severe tinnitus, compared to non-responders. We therefore increased the TFI score required for eligibility to 32 or higher at both screening and baseline and stratified the randomization to ensure equal numbers of OTO-313 and placebo patients within the ≥ 32 to ≤ 53 and ≥ 54 to 100 strata. The inclusion of these stratification factors (time since tinnitus onset and baseline TFI scores) allowed for a more in-depth evaluation of treatment benefit of OTO-313.

## Materials and methods

### Study design and patients

This randomized, double-blind, placebo-controlled, Phase 2 study was conducted in the United States, Germany, Poland, and the United Kingdom. This study was registered on ClinicalTrials.gov (NCT04829214) and conducted in compliance with applicable regulatory requirements, the Declaration of Helsinki, Good Clinical Practice guidelines, and central and local Ethics Committees and Institutional Review Boards. All participants provided written informed consent before enrollment.

Eligible patients were male or female aged 18–75 years, inclusive, with subjective unilateral tinnitus. Patients had to be consistently aware of their tinnitus throughout much of the waking day. The tinnitus had to be deemed by the investigator to be likely of cochlear origin (e.g., associated with acute hearing loss from noise trauma, barotrauma, blast trauma, middle ear surgery, age-related hearing loss, resolved otitis media, ototoxic drug exposure) with an onset of tinnitus of 2–12 months prior to signing informed consent. Tinnitus associated with COVID-19 infection or vaccination did not qualify for the study. TFI scores at both Screening and Baseline visits were required to be ≥ 32 for eligibility. Study patients could have audiometrically defined normal hearing or up to moderately severe hearing loss in the affected ear (study ear) as characterized by pure tone average of ≤ 70 dB at 1000, 2000, and 4000 Hz at Screening. Patients had to be able to use a smart phone diary to complete daily tinnitus ratings and complete at least five of the last 7 days of diary entries during the 14-days Lead-in period for eligibility. Exclusion criteria included Meniere’s disease. pulsatile tinnitus, tinnitus resulting from traumatic head or neck injury, any ongoing therapy known to be potentially tinnitus-inducing, severe or untreated depression or anxiety, and systemic or intratympanic steroids within 6 weeks prior to Screening, active middle ear disease including: abnormality or perforation of the tympanic membrane. Stable prior treatments (≥ 1 month prior to Screening) of antidepressant and anti-anxiety medications, over-the-counter supplements or medications for tinnitus, or use of hearing aids, noise generators, sound therapy devices, and any behavioral therapy for tinnitus were allowed but expected to continue for the duration of the study.

### Placebo response mitigation training

To mitigate placebo response, patients viewed a training video at screening entitled: “What It Means to Participate in Clinical Research Studies”, which explained the role of research subjects in a clinical trial, what to expect from clinical site staff, and the importance of randomization and placebo in a controlled trial. Prior to site activation, investigators, study coordinators, and audiologists underwent training on placebo response which included a discussion of factors affecting placebo response and best practices of how to interact with trial patients (e.g., neutral interactions with modest rapport, avoiding discussion of expected positive or negative outcomes) to minimize the risk of high placebo response.

### Randomization and treatment

Patients were randomized 1:1 to receive either 0.32 mg OTO-313 (1.6 mg/mL solution of gacyclidine in medium chain triglycerides) or placebo (solution of medium chain triglycerides). There were no differences in viscosity or appearance between OTO-313 and placebo. Randomized patients received a single (0.2 mL volume) intratympanic injection of either OTO-313 or placebo to the affected (study) ear at the Baseline visit. Patients remained recumbent for at least 15 min after the injection. To ensure approximately equal numbers of patients between OTO-313 and placebo, randomization was stratified by study site, duration of tinnitus (≥ 2 to ≤ 6 months since onset, > 6 to ≤ 12 months since onset), and the average TFI score at Screening and Baseline (≥ 32 to ≤ 53 points, ≥ 54 to ≤ 100 points). Patients were evaluated over a 16-weeks post-treatment period and returned for clinical site visits at Weeks 4, 8, 12, and 16.

### Efficacy evaluation

Efficacy assessments included the TFI (completed at Screening, Baseline (Day 1), Weeks 4, 8, 12, and 16), and the Patient Global Impression of Change to evaluate overall “global” tinnitus status as perceived by the patient (completed at Weeks 4, 8, 12, and 16). Daily ratings of tinnitus loudness and tinnitus annoyance utilizing numeric rating scales from 0 to 10 were completed each day between 6 p.m. and midnight using a smart phone.

### Safety evaluation

Safety data were collected at all visits and included collection and evaluation of treatment-emergent adverse events, vital signs, clinical laboratory measurements, otoscopic examinations, audiograms, tympanometry, concomitant medications, and Columbia-Suicide Severity Rating Scale assessments.

### Statistical considerations

The primary endpoint for this study was the percentage of responders for OTO-313 compared to placebo, where response was defined as achieving at least a 13-point reduction from baseline in the TFI score at both Weeks 4 and 8. It was observed from the Phase 1/2 study that the response rate, using this same definition, was 43% for OTO-313 (0.32 mg) and 13% for placebo [16]. Based on these results, the assumed rate of response was 43% for OTO-313 and 18% for placebo, which was the 13% observed response rate in the previous study plus 5% inflation for potential placebo response.

Assuming a 2-sided test and level of significance of 0.05, 60 patients in the placebo group and 60 patients in the OTO-313 group provided approximately 85% power to detect a treatment difference in favor of OTO-313. A discontinuation rate of 15% was projected, hence the total sample size for this 2-arm study was planned to be 140 patients, 70 subjects per arm. These sample size calculations were conducted using EASTv6.5 software with a test for a difference in proportions between two groups.

The primary endpoint was analyzed using the Mantel–Haenszel test controlling for duration of tinnitus and baseline TFI score. The change from baseline in tinnitus loudness, tinnitus annoyance, TFI total score, and each TFI subscale score was analyzed using a linear mixed-effects model with treatment, study day, treatment-by-study day interaction, gender, and tinnitus duration as fixed effects. PGIC was analyzed using a Cochrane-Mantel–Haenszel mean score test controlling for baseline TFI score and duration of tinnitus.

## Results

**Fig. 1:**
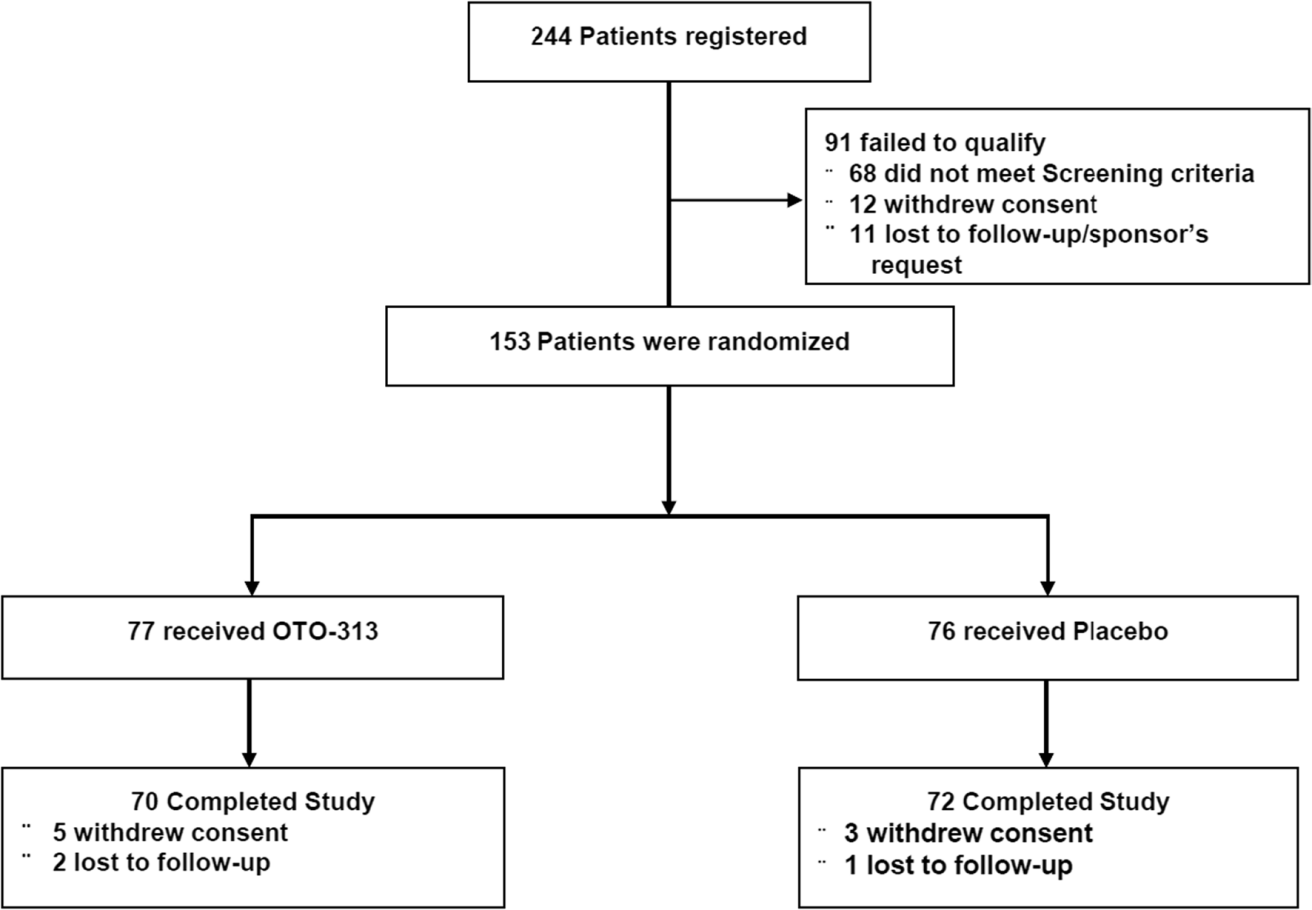
Enrollment

A total of 153 patients were randomized into the study with 77 receiving OTO-313 and 76 receiving placebo (Fig. 1). The most common reason for exclusion was not meeting Screening criteria, specifically not having a TFI score ≥ 32 at Screening and Baseline. Seven OTO-313 patients and 4 placebo patients either withdrew consent or were lost to follow-up, and therefore did not complete the study. Due to investigator error, five participants not meeting onset of tinnitus of 2–12 months were erroneously included in the randomization. One participant had tinnitus for less than 2 months and was included in the acute tinnitus group and four participants had tinnitus greater than 12 months (14–21 months) and were included in the chronic tinnitus group. Once randomized, the primary analysis was based on intent-to-treat and all were included in the analysis. Most patients were randomized in the United States (*N* = 114), followed by Poland (*N* = 25), Germany (*N* = 13), and the United Kingdom (*N* = 1).

Demographics were well balanced across OTO-313 and placebo groups with respect to sex, age, race, and ethnicity (Table 1). Baseline characteristics related to tinnitus were also similar between OTO-313 and placebo groups including average months since tinnitus onset (6.6 months for OTO-313 versus 6.7 months for placebo), category of tinnitus duration (≥ 2 to ≤ 6 months: 42.9% for OTO-313 versus 43.4% for placebo; > 6 to ≤ 12 months: 57.1% for OTO-313 versus 56.6% for placebo), mean baseline TFI scores (62.6 for OTO-313 versus 65.2 for placebo), and baseline TFI category (≥ 32 to ≤ 53 points: 27.3% for OTO-313 versus 30.3% for placebo; ≥ 54 to ≤ 100 points: 72.7% for OTO-313 versus 69.7% for placebo). As indicated, a larger number of study patients had severe tinnitus compared to those with more moderate tinnitus. With respect to etiology of tinnitus as determined by the investigators, the majority of patients (93.5% of OTO-313 patients, 90.8% of Placebo patients) had tinnitus that was associated with hearing loss at least at the time of tinnitus initiation and was perhaps transient in some patients, since hearing as assessed during the Baseline visit was characterized as normal (≤ 25 dB) in 57.1% of OTO-313 patients and in 55.3% of placebo patients.

**Table 1:** Patient demographics and baseline characteristics

	OTO-313 (*N* = 77)	Placebo (*N* = 76)
Sex, *n* (%)		
Male	36 (46.8)	38 (50.0)
Female	41 (53.2)	38 (50.0)
Age in years, mean (SD)	52.4 (12.8)	50.8 (15.2)
Race, *n* (%)		
White	67 (87.0)	68 (89.5)
Black or African American	6 (7.8)	5 (6.6)
American Indian or Alaska Native	0	1 (1.3)
Asian	1 (1.3)	0
Other	3 (3.9)	2 (2.6)
Ethnicity, *n* (%)		
Hispanic or Latino	8 (10.4)	7 (9.2)
Not Hispanic or Latino	67 (87.0)	68 (89.5)
Not reported	2 (2.6)	1 (1.3)
Unknown	0	0
Months since tinnitus onset, mean (SD) [range]	6.6 (3.5) [2.0–15.0]	6.7 (3.8) [0–21.0]
Duration of tinnitus, *n* (%)^a^		
≥ 2 to ≤ 6 months	33 (42.9)	33 (43.4)
> 6 to ≤ 12 months	44 (57.1)	43 (56.6)
Baseline TFI overall score, mean (SD) [range]^b^	62.6 (13.8) [33.6–97.4]	65.2 (15.5) [34.2–99.2]
Category of baseline TFI overall score, *n* (%)		
≥ 32 to ≤ 53 points	21 (27.3)	23 (30.3)
≥ 54 to ≤ 100 points	56 (72.7)	53 (69.7)
Etiology of tinnitus, *n* (%)		
Associated with sensorineural hearing loss	56 (72.7)	44 (57.9)
Acute hearing loss from noise or other trauma	13 (16.9)	21 (27.6)
Age-related hearing loss	3 (3.9)	4 (5.3)
Resolved otitis media	4 (5.2)	6 (7.9)
Ototoxic drug exposure	0	1 (1.3)
Other	7 (9.1)	10 (13.2)
Hearing loss of treated ear at baseline, *n* (%)		
Normal hearing: ≤ 25 dB	44 (57.1)	42 (55.3)
Mild hearing loss: 26–40 dB	20 (26.0)	20 (26.3)
Moderate hearing loss: 41–55 dB	11 (14.3)	9 (11.8)
Moderately-severe hearing loss: 56–70 dB	2 (2.6)	3 (3.9)

^a^Subjects whose duration of tinnitus was > 12 months was collapsed in with > 6 to ≤ 12 months category

^b^Baseline TFI overall score is defined as the average of the TFI overall scores from both the Screening and the Day 1 Visits

### Efficacy

**Fig. 2:**
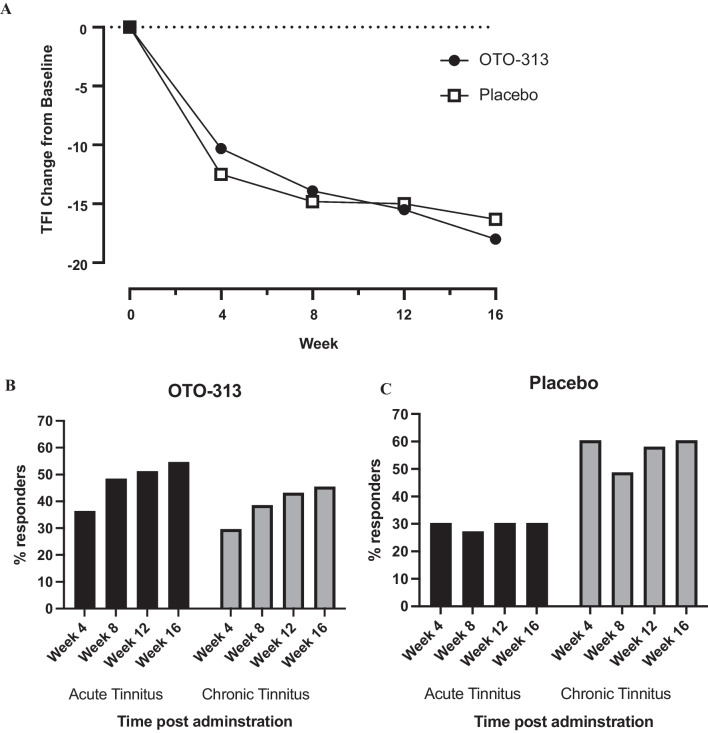
**A** Mean change from baseline in TFI scores. Percentage of participants with acute and chronic tinnitus responding to **B** OTO-313 and **C** Placebo

Treatment with OTO-313 did not meet the primary efficacy endpoint in this study. At each visit OTO-313 and placebo produced a similar percentage of responders on the TFI (≥ 13-points, considered to be clinically meaningful [19]). At week 8, the primary endpoint of the study, a clinically meaningful change was observed for 26% of OTO-313 participants versus 36% for placebo (Table 2). The mean change from baseline on the TFI was also reduced for both OTO-313 and placebo groups to a similar degree at Weeks 4, 8, 12, and 16 demonstrating a high placebo response and benefit for both groups (Fig. 2A).

**Table 2:** Number of TFI responders (patients with ≥ 13-point reduction on the TFI) following treatment with OTO-313 or placebo

Week	OTO-313 (*N* = 77) *n* (%)	Placebo (*N* = 76) *n* (%)	*p* value
4	25 (33%)	36 (47%)	0.122
8	33 (43%)	30 (40%)	0.916
Week 4 and 8*	20 (26%)	27 (36%)	0.385
12	36 (47%)	35 (46%)	0.935
16	38 (49%)	36 (47%)	0.835

^*^Primary endpoint of the study was number of TFI responders at both Week 4 and 8

When stratified by tinnitus duration, OTO-313 and placebo groups in both strata exhibited similar mean TFI scores at baseline. At post-dose visits at Weeks 4, 8, 12, and 16 for the ≥ 2 months to ≤ 6 months stratum, change from baseline in TFI scores and percent TFI responders favored OTO-313 versus placebo with significantly more responders treated with OTO-313 than placebo at Week 16 (Table 3, Fig. 2B and C). For the > 6 months to ≤ 12 months stratum, however, change from baseline in TFI scores and percent TFI responders favored placebo over OTO-313 treatment at each post-dose assessment, with significantly more placebo responders at Week 4 (Table 3, Fig. 2B and C).

**Table 3:** Effect of OTO-313 or placebo on mean TFI, change from baseline in TFI, and number of responders on TFI: stratification by tinnitus duration

Study visit	Tinnitus duration ≥ 2 to ≤ 6 months			Tinnitus duration > 6 to ≤ 12 months		
	OTO-313 (*N* = 33)	Placebo (*N* = 33)	*p* value	OTO-313 (*N* = 44)	Placebo (*N* = 43)	*p* value
Baseline						
TFI mean (SD)	63.9 (15.7)	61.8 (18.2)		61.9 (14.6)	68.3 (12.7)	
Week 4			0.712			0.006
TFI mean (SD)	51.5 (19.3)	54.8 (19.0)		53.5 (16.3)	52.2 (20.6)	
CFB mean (SD)	− 12.5 (16.4)	− 7.5 (15.4)		− 8.7 (11.9)	− 16.5 (16.5)	
#Responders (%)	12 (36.4)	10 (30.3)		13 (29.6)	26 (60.5)	
Week 8			0.064			0.498
TFI mean (SD)	47.3 (22.0)	52.5 (22.6)		50.7 (16.8)	48.8 (23.4)	
CFB mean (SD)	− 16.6 (18.6)	− 8.9 (19.1)		− 12.0 (15.5)	− 19.4 (21.9)	
#Responders (%)	16 (48.5)	9 (27.3)		17 (38.6)	21 (48.8)	
Week 12			0.102			0.290
TFI mean (SD)	45.7 (23.4)	52.8 (22.8)		48.6 (17.8)	49.8 (23.1)	
CFB mean (SD)	− 18.6 (20.2)	− 9.8 (20.3)		− 13.6 (17.6)	− 18.8 (20.6)	
#Responders (%)	17 (51.2)	10 (30.3)		19 (43.2)	25 (58.1)	
Week 16			0.029			0.228
TFI mean (SD)	43.4 (23.6)	48.1 (26.7)		45.6 (19.7)	49.5 (24.2)	
CFB mean (SD)	− 20.2 (20.1)	− 12.6 (23.1)		− 16.5 (19.9)	− 19.0 (22.5)	
#Responders (%)	18 (54.6)	10 (30.3)		20 (45.5)	26 (60.5)	

Baseline TFI scores were averaged between the Screening and Baseline visits

*CFB* change from baseline TFI score

#Responders indicates number of patients with ≥ 13-pt reduction from baseline on the TFI

As stated earlier, 109 patients in this study had TFI scores in the ≥ 54- to 100-point range, while 46 patients had TFI scores in the more moderate range of ≥ 32- to ≤ 53-points. Over the course of the study in both strata, mean TFI scores, change from baseline in TFI scores, and percent TFI responders were similar between OTO-313 and placebo groups (Table 4).

**Table 4:** Effect of OTO-313 or placebo on mean TFI, change from baseline in TFI, and number of responders on TFI: stratification by baseline TFI score

Study visit	Baseline TFI score range ≥ 32 to ≤ 53 points		Baseline TFI score range ≥ 54 to 100 points	
	OTO-313 (N = 21)	Placebo (N = 23)	OTO-313 (N = 56)	Placebo (53)
Baseline				
Mean (SD)	46.0 (6.1)	47.4 (5.7)	69.0 (10.1)	73.3 (11.3)
Week 4				
TFI mean (SD)	38.4 (9.6)	41.4 (12.1)	57.6 (17.0)	58.5 (20.3)
CFB mean (SD)	− 7.7 (9.1)	− 6.0 (10.6)	− 11.2 (15.3)	− 15.4 (17.9)
#Responders (%)	4 (19.0)	9 (39.1)	21 (37.5)	27 (50.9)
Week 8				
TFI mean (SD)	36.4 (12.8)	39.9 (12.7)	53.5 (18.9)	55.4 (25.0)
CFB mean (SD)	− 8.6 (13.6)	− 7.6 (11.8)	− 15.6 (17.5)	− 18.0 (23.8)
#Responders (%)	7 (33.3)	7 (30.4)	26 (46.4)	23 (43.4)
Week 12				
TFI mean (SD)	35.2 (13.1)	41.6 (15.7)	51.4 (20.5)	55.0 (24.3)
CFB mean (SD)	− 9.9 (14.8)	− 6.1 (15.2)	− 17.3 (19.5)	− 18.7 (21.8)
#Responders (%)	7 (33.3)	6 (26.1)	29 (51.8)	29 (54.7)
Week 16				
TFI mean (SD)	30.8 (12.9)	37.5 (16.3)	49.3 (21.6)	54.1 (26.7)
CFB mean (SD)	− 14.2 (14.8)	− 10.0 (13.8)	− 19.3 (21.3)	− 19.1 (25.4)
#Responders (%)	9 (42.9)	10 (43.5)	29 (51.8)	28 (52.8)

Baseline TFI scores were averaged between the screening and baseline visits

*CFB* change from baseline TFI score

#Responders indicates number of patients with ≥ 13-pt reduction from baseline on the TFI

**Fig. 3:**
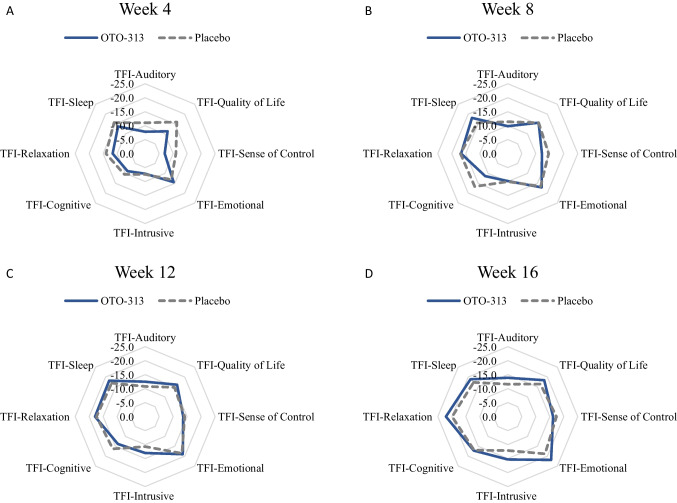
Radar plots of change from baseline in TFI subscale scores by treatment and visit. The further from the center the spike is the larger the response

Radar plots of the TFI subscales indicate an impact on multiple TFI subdomains for both OTO-313 and placebo groups (Fig. 3), suggesting no single subscale drove the overall improvement on the TFI. Interestingly, at Week 16 a trend for treatment benefit in favor of OTO-313 was apparent across several subdomains; this result is consistent with the trend for a reduction for OTO-313 versus placebo in mean change from baseline at Week 16 for overall TFI scores (Fig. 2), and with the improvement with OTO-313 treatment in the ≥ 2- to ≤ 6-month tinnitus stratum (Table 3).

**Fig. 4:**
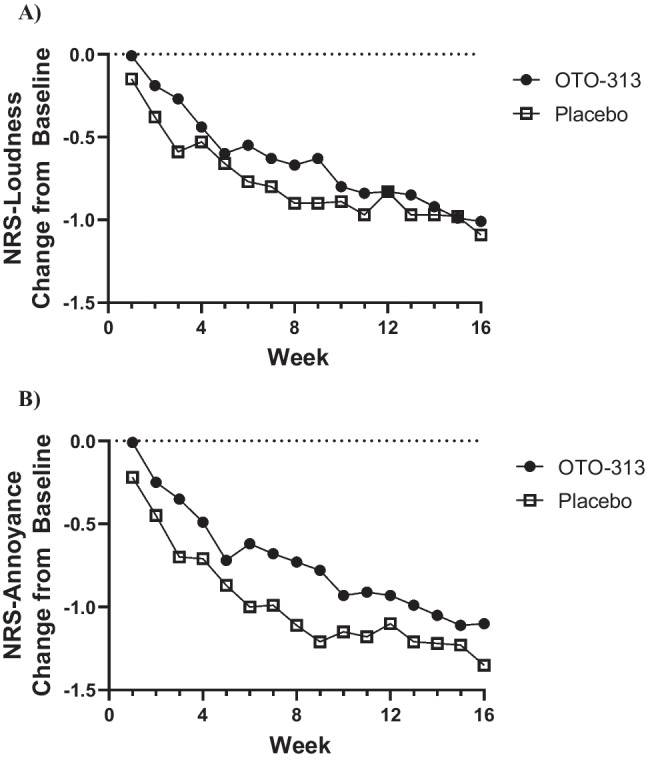
Effect of OTO-313 on **A** Tinnitus loudness and **B** Tinnitus annoyance

Baseline tinnitus loudness and tinnitus annoyance as captured by daily diaries were similar between OTO-313 and placebo groups (loudness [mean (SD)]: OTO-313, 7.1 (1.7) units; Placebo, 7.6 (1.6) units; annoyance [mean (SD)]: 6.9 (1.7) units; Placebo, 7.5 (1.8) units). Decreases from baseline in tinnitus loudness and annoyance were observed for both OTO-313 and placebo groups (Fig. 4). By Week 16, the mean changes in both scales were approximately 1.0–1.3 units on the 0–10 numeric rating scale which is generally considered clinically meaningful. Overall compliance for completing the daily diary was good, averaging 92.6% across the 16 weeks of follow-up. High compliance was likely due to the brief nature of the diary (only 2 questions), utilization of the patient’s own device, the requirement to complete the diary at the same time of day, and by the programmed reminders.

Similar numbers of OTO-313 and placebo patients reported an improvement (i.e., minimally improved, much improved, or very much improved) on the PGIC at Week 4, 8, 12, and 16 (Table 5). The most selected response category on the PGIC was “unchanged” for both OTO-313 and placebo groups (Table 5).

**Table 5:** PGIC scores following treatment with OTO-313 or placebo

	OTO-313	Placebo
	(*N* = 77)	(*N* = 76)
	*n* (%)	*n* (%)
Week 4		
Very much improved	0	2 (2.6)
Much improved	10 (13.0)	4 (5.3)
Minimally improved	15 (19.5)	18 (23.7)
Unchanged	33 (42.9)	40 (52.6)
Minimally worse	12 (15.6)	6 (7.9)
Much worse	4 (5.2)	2 (2.6)
Very much worse	0	1 (1.3)
Week 8		
Very much improved	4 (5.2)	5 (6.6)
Much improved	4 (5.2)	5 (6.6)
Minimally improved	19 (24.7)	18 (23.7)
Unchanged	31 (40.3)	31 (40.8)
Minimally worse	6 (7.8)	8 (10.5)
Much worse	4 (5.2)	3 (3.9)
Very much worse	0	0
Week 12		
Very much improved	2 (2.6)	4 (5.3)
Much improved	8 (10.4)	6 (7.9)
Minimally improved	18 (23.4)	13 (17.1)
Unchanged	30 (39.0)	37 (48.7)
Minimally worse	6 (7.8)	6 (7.9)
Much worse	5 (6.5)	4 (5.3)
Very much worse	1 (1.3)	1 (1.3)
Week 16		
Very much improved	7 (9.1)	4 (5.3)
Much improved	5 (6.5)	9 (11.8)
Minimally improved	15 (19.5)	10 (13.2)
Unchanged	29 (37.7)	42 (55.3)
Minimally worse	9 (11.7)	3 (3.9)
Much worse	4 (5.2)	2 (2.6)
Very much worse	0	1 (1.3)

### Safety

A single intratympanic injection of OTO-313 was well-tolerated. The number and percentage of adverse events overall was similar between patients who received OTO-313 and patients who received placebo (Table 6). Most adverse events were associated with the ear or with a non-ear-related infection. Most adverse events were mild or moderate in intensity and no subjects discontinued the study due to an adverse event. There were two serious adverse events (one was atherosclerosis and the other was exacerbation of ischemic heart disease), both of which were not considered related to study drug, and which resolved during the study with both patients completing the study. There was one pinhole perforation in a patient that received OTO-313 at the Week 4 visit, which resolved by Week 8. There were no clinically significant changes in vital signs (pulse, blood pressure), clinical laboratory measures, otoscopy, tympanometry, audiometry, or assessment of suicidality.

**Table 6:** Adverse events occurring in ≥ 2 patients in either treatment group

Body system organ class Adverse event (AE)	OTO-313 (*N* = 77) *n* (%)	Placebo (*N* = 74) *n* (%)	Total (*N* = 151) *n* (%)
Number of subjects with at least 1 AE reported	32 (41.6)	30 (40.5)	62 (41.1)
Ear disorders	11 (14.3)	13 (17.6)	24 (15.9)
Tinnitus	8 (10.4)	8 (10.8)	16 (10.6)
Vertigo	2 (2.6)	1 (1.4)	3 (2.0)
Infections	11 (14.3)	13 (17.6)	24 (15.9)
COVID-19	5 (6.5)	7 (9.5)	12 (7.9)
Nasopharyngitis	2 (2.6)	0	2 (1.3)
Urinary tract infection	2 (2.6)	0	2 (1.3)
Psychiatric disorders	3 (3.9)	2 (2.7)	5 (3.3)
Insomnia	3 (3.9)	1 (1.4)	4 (2.6)

*n* = number of people that reported the adverse event

% = *n* divided by the total number of people in the column total × 100

## Discussion

Tinnitus is a widely prevalent and often disabling condition with existing interventions that focus on sound and behavioral therapies that do not address the underlying pathology. OTO-313 is a sustained exposure formulation of the high-affinity NMDA receptor antagonist gacyclidine which is intended to block the heightened activation of cochlear NMDA receptors that may underly the generation of tinnitus. The superiority of OTO-313 over placebo demonstrated in the initial Phase 1/2 study in a relatively small sample [16], was not confirmed in this larger, well-powered Phase 2 study. Despite including measures to minimize placebo response, a high placebo response was observed which impacted the ability to consistently show an improvement with OTO-313 treatment.

Participants were characterized as having acute tinnitus if they experienced tinnitus for less than, or equal to, 6 months and chronic tinnitus if their duration was greater than 6 months. When divided into these groups it was expected that the acute group results would resemble that of our previous Phase 1/2 trial results [16]. Indeed, the percentage of responders to OTO-313 was similar in the acute participants and numerically favored OTO-313 compared to placebo, although the percentage of responders to the placebo was double that seen in the earlier study. The percentage of responders to OTO-313 in the chronic group was lower than for the acute group, as anticipated, but did show an increase in response rate, as seen in the acute group, over time. The greatest anomaly compared to the previous trial was in the percentage of respondents to the placebo in the chronic group. The response rate to the same placebo was twice as high (60%) compared to the placebo response in the acute group (30%). This result had a large effect on the group statistics. With OTO-313 treatment, the number of responders grew with time, consistent with an expected therapeutic response. In contrast, the placebo response did not demonstrate this growth over time, consistent with the non-specific nature of the placebo.

The difference in placebo response between the two duration groups is not easily explained. Tinnitus is highly heterogenous, although participants were randomly assigned to treatment groups it is possible that some variable that was not accounted for, distinguished the chronic placebo group from the acute group. Another possibility is that the chronic group, having suffered from tinnitus for a longer period compared to the acute group, had heightened expectations for a benefit. Chronic tinnitus sufferers experience ongoing worries that can maintain or exacerbate the condition [20]. Expectations of a treatment benefit and placebo response have been noted in placebo-controlled trials for several subjective conditions including depression and chronic pain [21, 22]. The expectancy theory holds that a reduction in symptoms may occur from receiving a treatment (whether it’s active or placebo) or from participating in a clinical trial in which the patient interacts with healthcare providers or undergoes medical procedures. Evidence of patient characteristics influencing placebo response is limited and duration of disease or condition has not been evaluated in placebo response. Results from antidepressant trials demonstrate that placebo response decreases with increasing severity of baseline depression scores [21]. However, this did not seem to be a factor in the present study since baseline TFI scores were similar between acute and chronic tinnitus groups.

Steps were taken to minimize placebo response including patient, investigator, and site staff training. Patients watched a video about their role in clinical research, how it differs from receiving standard medical care, and the purpose of placebo. Investigators and site staff were trained on the scientific basis of placebo response and best ways to communicate neutrally to study patients to minimize placebo response. In addition, the 1:1 randomization ratio was selected in part to minimize placebo response as patients were informed they had a 50/50 chance of receiving active or placebo. Unbalanced randomization in which the likelihood of receiving active is higher than 50% has been associated with higher placebo response rates [23]. Additional design changes could be considered in future studies to limit placebo response including a placebo lead-in period in which all patients receive a placebo injection and only those with a limited response to placebo would then be randomized to either placebo or active. This lengthens the duration of the study and exposes patients to more than one injection, but a worthwhile consideration to evaluate a drug response more effectively.

Considering the OTO-313 results alone there was not a large difference in responders between acute and chronic groups. It was predicted that the cochlear locus of action of the drug would diminish its effectiveness with tinnitus becoming chronic, due to a centralization effect [17, 18]. The observation of 10% more self-reported acute tinnitus participants responding to OTO-313 compared to chronic tinnitus participants is not convincing evidence for a therapeutic window at the dose tested. It is possible that a therapeutic window does exist, but it may be very close to tinnitus onset, potentially less than the 2 months used as an inclusion criterion in this study. If the therapeutic window is very narrow the ability to diagnose and then treat tinnitus using the approach tested would be difficult in the time available. The therapy would rely on patients presenting close to onset of tinnitus and then being referred for therapy. This presents a research and health service delivery challenge as tinnitus can be of short duration and spontaneously resolve.

Several NMDA receptor antagonists have been evaluated in the treatment of tinnitus, each with limited success. This includes intratympanic administration of OTO-313 (evaluated in this study) and AM-101, a hyaluronic acid formulation of S-ketamine [24], oral administration of neramexane [25], and inhalation of nitrous oxide [26]. As with OTO-313, AM-101 was primarily examined in acute tinnitus, while both neramexane and nitrous oxide were tested in more chronic tinnitus. We considered OTO-313 a superior approach because of gacyclidine’s high affinity and selectivity for NMDA receptors [12], relative to most of the other agents in the class, and its favorable inner ear pharmacokinetic properties, at least in animals [14]. OTO-313 is still the agent of choice, in our view, although changes to the study design to account for high placebo response must be considered in future studies. Based on animal studies, the 0.32 mg dose of OTO-313 administered was projected to provide sustained exposures of gacyclidine to the cochlea at pharmacologically relevant levels for several weeks. It is difficult to know whether inner ear concentrations are similar between rats and humans following intratympanic administration of OTO-313 as an assessment of this nature in humans is prohibitively invasive. Evaluation of a higher dose of OTO-313 may be worthwhile assuming the placebo response can be mitigated.

In conclusion, the results from this study conflict with findings from the earlier Phase 1/2 study, primarily in that there was a much larger than anticipated placebo response. Considerations for any future studies include incorporation of designs to minimize placebo response and targeting acute tinnitus within 6 months or less since onset.

## Data Availability

Due to the commercial nature of the research supporting data is not available.
